# Development a prediction model for identifying bacterial meningitis in young infants aged 29–90 days: a retrospective analysis

**DOI:** 10.1186/s12887-022-03813-1

**Published:** 2023-02-09

**Authors:** Jiahui Wu, Ting Shi, Yongfei Yue, Xiaoxing Kong, Fangfang Cheng, Yanqun Jiang, Yuanxi Bian, Jianmei Tian

**Affiliations:** 1 Department of Infectious Diseases, Children’s Hospital of Soochow University, No. 92, Zhongnan Street, Suzhou, 215025 China; 2 Department of Obstetrics and Gynecology, The Affiliated Suzhou Hospital of Nanjing Medical University, Suzhou Municipal Hospital, Suzhou, China

**Keywords:** Prediction, Young infants, Bacterial meningitis, Cerebrospinal fluid pleocytosis

## Abstract

**Background:**

The early diagnosis and treatment of bacterial meningitis (BM) in young infants was very critical. But, it was difficult to make a definite diagnosis in the early stage due to nonspecific clinical symptoms. Our objectives were to find the risk factors associated with BM and develop a prediction model of BM especially for young infants.

**Methods:**

We retrospectively reviewed the clinical data of young infants with meningitis between January 2011 and December 2020 in Children’s Hospital of Soochow University. The independent risk factors of young infants with BM were screened using univariate and multivariate logistic regression analyses. The independent risk factors were used to construct a new scoring model and compared with Bacterial Meningitis Score (BMS) and Meningitis Score for Emergencies (MSE) models.

**Results:**

Among the 102 young infants included, there were 44 cases of BM and 58 of aseptic meningitis. Group B *Streptococcus* (22, 50.0%) and *Escherichia coli* (14, 31.8%) were the main pathogens of BM in the young infants. Multivariate logistic regression analysis identified procalcitonin (PCT), cerebrospinal fluid (CSF) glucose, CSF protein as independent risk factors for young infants with BM. We assigned one point for CSF glucose ≤ 1.86 mmol/L, two points were assigned for PCT ≥ 3.80 ng/ml and CSF protein ≥ 1269 mg/L. Using the not low risk criterion (score ≥ 1) with our new prediction model, we identified the young infantile BM with 100% (95% CI 91.9%-100%) sensitivity and 60.3% (95% CI 46.4%-72.9%) specificity. Compared with BMS and MSE model, our prediction model had larger area under receiver operating characteristic curve and higher specificity, the differences were statistically significant.

**Conclusion:**

Our new scoring model for young infants can facilitate early identification of BM and has a better performance than BMS and MSE models.

## Background

Bacterial meningitis (BM) is a life-threatening bacterial infection, with the highest incidence reported in young infants [1]. Due to the low resistance of young infants, and the blood–brain barrier has not been fully developed, the bacteria are easy to reach the meninges through the blood–brain barrier to cause infection of the central nervous system. But, diagnostic signs for BM in infants are nonspecific, they do not often exhibit the general symptoms and may only be fevered or look unwell [2, 3]. It is important for young infantile BM to early diagnose and properly manage to reduce the mortality and complication.

Physicians have been trained to administer antibiotics for infants suspected bacterial infection as soon as possible. If antibiotics need to be given before the lumbar puncture (LP) is performed, the antibiotics can sterilize the cerebrospinal fluid (CSF) making it less likely that bacteria will grow [4]. This can cause difficulty in confirming the diagnosis of BM, especially if there are other abnormalities in the CSF such as pleocytosis. Several models have been developed to predict BM [5–9], which can aid physician in their diagnostic approach. However, none of the existing models performed well enough to recommend as routine use in individual patient management which might be attributed to the wide range of applicable ages of the models. Nigrovic et al. identified a classic Bacterial Meningitis Score (BMS) model [5], but this model misclassified a few infants aged ≤ 60 days with BM as being at low risk for the disease [10], also it had low specificity and should not be applied clinical use to young infants [11]. Therefore, our objective was to generate a new scoring model for young infants (29–90 days) suspected BM who performed LP and had CSF pleocytosis. Moreover, we assessed the role of BMS model [5] and Meningitis Score for Emergencies (MSE) model [9] in our study population.

## Materials and methods

### Patients

We retrospectively reviewed the clinical records of young infants aged from 29 to 90 days with suspicions of meningitis (the ICD code of initial diagnosis was G04.913), and patients in whom a LP was accepted and with CSF pleocytosis (CSF leukocyte count > 10 × 10^6^ /L). In our study, young infants suspected of meningitis and who have completed LP must have the following examinations: peripheral blood cell count, peripheral blood inflammatory markers (C-reactive protein CRP, procalcitonin PCT), CSF cell count, CSF glucose and protein, CSF Gram stain, CSF and peripheral blood culture, cranial magnetic resonance imaging or computerized tomography. It was also suggested to conduct CSF virus detection or CSF metagenomics next generation sequencing if conditions permit. We excluded those patients who were not previously healthy (with the history of severe neurological disease or ventricular drainage or primary immune-deficiencies), underwent a traumatic LP (> 1000 × 10^6^ /L red blood cells in the CSF), diagnosed with a definite viral meningitis (such as enteroviral meningitis, herpes simplex viral meningitis), or treated with antibiotics within 72 h before the diagnostic LP. We also excluded the cases with incomplete clinical data. Then, we selected eligible patients who were finally diagnosed as BM (the ICD code was G00.900) or Aseptic meningitis (AM, the ICD code was G03.001). The eligible patients were from Children’s Hospital of Soochow University between January 2011 and December 2020. The study was approved by the Ethics Committee of Children’s Hospital of Soochow University.

Infantile BM was diagnosed according to either one of the following two criteria: (1) the CSF culture was positive for a bacterial pathogen (*Streptococcus pneumonia*, group B *Streptococcus* (GBS), *Escherichia coli*, *Staphylococcus aureus*, *Neisseria meningitidis*, *Haemophilus influenza*, etc.); or (2) the presence of CSF pleocytosis and with a positive blood culture. Organisms (such as *coagulase-negative staphylococci, Propionobacterium acnes, Streptococcus viridans, Corynebacterium spp, and other diphtheroids*) cultured in previously healthy patients were considered to be contaminants. AM was defined as CSF pleocytosis with negative bacterial cultures of the CSF and blood. In our study, AM also include the presence of negative viral tests if performed. Complications of acute meningitis included seizure, subdural effusion, hydrocephalus, brain abscess, suspected ventriculitis and cerebral infarcts.

### Data collection

We collected information about demographic, clinical, laboratory characteristics, and LP results. Demographic and clinical characteristics included age, gender, occurrence of seizures, anterior fontanel pressure (AFP). The presence of seizures and increased AFP were determined by two treating physicians. The laboratory values were obtained closest before the time to the LP, the data included peripheral white blood cell (WBC) count, peripheral absolute neutrophil count (ANC), CRP, PCT measurements, blood culture. LP results included CSF WBC count and CSF ANC, CSF glucose, CSF protein, CSF Gram stain, CSF culture.

### Statistical analyses

Statistical analyses were performed using SPSS 27.0. Our analysis showed that the measurement data in this study were not normally distributed. Therefore, the measurement data were expressed as medians (quartiles), and the count data were expressed as frequencies (percentages). Mann–Whitney test for measurement date and χ2 test for count date were used to compare variables between groups. We conducted a receiver operating characteristic (ROC) curve analysis including significant continuous variables selected by univariate analysis. Continuous variables were converted to dichotomous variables according to the optimal cutoff points used by the Youden index. Risk factor analysis was performed using univariate and multivariate logistic regression analyses. In multivariate logistic regression analysis, the forward stepwise method was used to select independent risk factors for young infants with BM. Hosmer–Lemeshow test was used to identify the fitness of the regression model. The score point of each predictor was determined by the value of logistic coefficient. Area under the ROC curve was calculated to evaluate capacity of the models. The sensitivity and specificity for each scoring models were calculated. Two-tailed analysis with *P* < 0.05 indicated that the difference was statistically significant.

## Results

### Main characteristics of patients

There were 133 infants aged from 29 to 90 days who had CSF pleocytosis and were initially diagnosed with meningitis, 27 cases with incomplete data were excluded. We also excluded 4 infants who were finally diagnosed with enteroviral meningitis. Eventually, the remaining 102 young infants including 44 (43.1%) with BM and 58 (56.9%) with AM were enrolled in our study. In the AM group, 32.8% (19/58) of the cases received Enterovirus testing, 77.6% (45/58) received Herpes simplex virus testing, and none of them had positive results. These children without positive pathogens were finally diagnosed as AM. Cases of BM were caused by the following pathogens: GBS (22, 50.0%), *Escherichia coli* (14, 31.8%), *Streptococcus pneumonia* (2, 4.5%), *Klebsiella pneumonia* (1, 2.3%), *Enterococcus species* (4, 9.1%), and *Staphylococcus aureus* (1, 2.3%). In the BM group, there were 6 infants with positive CSF Gram stain, and all of them were eventually cultured pathogenic bacteria. The bacterial pathogen was identified in both CSF and blood culture in 18 patients (40.9%), CSF culture alone in 16 patients (36.4%), and blood culture alone in 10 patients (22.7%). All the complications identified (37.9% of the patients) were seizures in AM group. Infants with BM could be combined with multiple complications rather than a single complication, the incidence of one or more complications was 59.1% in BM group. The main characteristics of the patients with BM and AM are shown in Table 1.

**Table 1:** Characteristics between bacterial meningitis patients and aseptic meningitis patients

Variable	Bacterial meningitis	Aseptic meningitis	Z or χ^2^ value	*P* value
Age in days, median (IQR)	59.5(32.5–74.5)	52.5(41–67.5)	0.16	0.874
Male, n(%)	26(59.1)	31(53.4)	0.32	0.570
Fever (T ≥ 37.3℃), n(%)	40(90.9)	58(96.6)	0.60	0.439
Seizure, n(%)	13(29.5)	22(37.9)	0.78	0.377
Increased AFP, n(%)	25(56.8)	26(44.8)	1.44	0.230
Complication, n(%)	26(59.1)	22(37.9)	4.50	0.034
Blood test results, median (IQR)				
WBC count, × 10^9^ /L	7.3(3.8–14.0)	11.8(7.7–16.0)	2.62	0.009
ANC, × 10^9^ /L	4.5(2.2–9.9)	5.2(3.5–10.0)	1.11	0.266
CRP, mg/L	90.1(63.7–163.2)	21.3(2.7–79.3)	4.27	< 0.005
PCT, ng/ml	9.5(3.8–23.6)	0.67(0.24–1.38)	4.04	< 0.005
CSF test results, median (IQR)				
WBC count, × 10^6^ /L	775(317–2153.8)	200(31.5–894)	3.51	< 0.005
ANC, × 10^6^ /L	503(103.3–1336.5)	45(10.5–523.75)	3.63	< 0.005
CSF glucose, mmol/L	1.8(0.7–2.5)	3.0(2.3–3.4)	4.95	< 0.005
CSF protein, mg/L	1730(1156.3–2933.3)	1071.5(731.3–1801.3)	3.63	< 0.005
Positive Gram stain, n(%)	6(13.6)	0(0)	6.12	0.013

*WBC* white blood cell, *ANC* absolute neutrophil count, *CRP* C-reactive protein, *PCT* procalcitonin, *AFP* anterior fontanel pressure, *CSF* cerebrospinal fluid, *IQR* interquartile range, *T* temperature

### Prediction model for young infants with BM

Meningitis associated clinical characteristics and laboratory parameters were compared by using univariate analysis, significant differences(*P* < 0.05) were demonstrated in peripheral WBC count, CRP, PCT, CSF WBC count, CSF ANC, CSF glucose, CSF protein and positive Gram stain between the BM and AM (Table 1). Optimum cutoff values for above significant continuous variables were determined by analyzing the ROC curve and Youden index. Then the following dichotomous variables were selected in a forward stepwise multivariable logistic regression analysis: male, history of seizure, increased AFP, peripheral WBC count ≤ 7.43 × 10^9^ /L, CRP ≥ 58.3 mg/L, PCT ≥ 3.80 ng/ml, CSF ANC ≥ 58.5 × 10^6^ /L, CSF glucose ≤ 1.86 mmol/L, CSF protein ≥ 1269 mg/L. PCT ≥ 3.80 ng/ml, CSF glucose ≤ 1.86 mmol/L and CSF protein ≥ 1269 mg/L were independent predictors of young infants with BM (Table 2). Hosmer–Lemeshow test was 0.42, which indicated a lack of deviation between the model and observed event rate, and the prediction model worked well. The area under the ROC curve of this regression model was 0.93 (95% confidence interval (CI) 0.88–0.98).

**Table 2:** Multivariable logistic regression analyses for prediction of young infants with bacterial meningitis

Variables	Logistic coefficient (β)	Odd ratio (95%CI)	*P* value
PCT ≥ 3.80 ng/ml	2.69	14.66(4.10–52.50)	< 0.005
CSF glucose ≤ 1.86 mmol/L	1.81	6.09(1.50–24.67)	0.011
CSF protein ≥ 1269 mg/L	2.42	11.26(2.95–42.96)	< 0.005

*PCT* procalcitonin, *CSF* cerebrospinal fluid, *CI* confidence interval

**Fig. 1:**
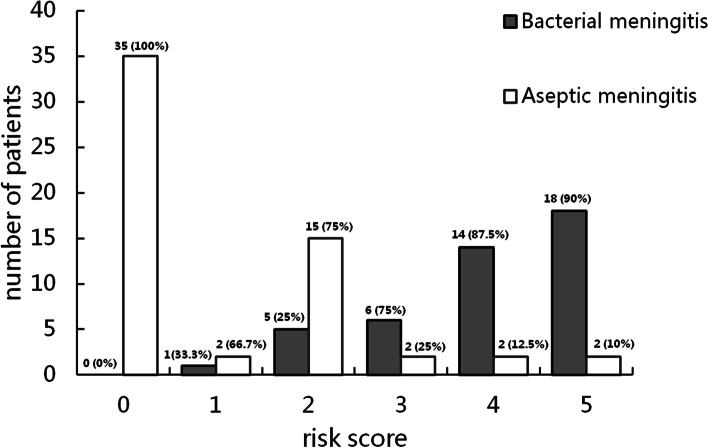
Distribution of young infants with bacterial and aseptic meningitis by our new scoring model

We developed a new scoring model for young infants with BM based on the logistic coefficient of significant predictors, two points were assigned to PCT ≥ 3.80 ng/ml and CSF protein ≥ 1269 mg/L, one point to CSF glucose ≤ 1.86 mmol/L. The range of the resulting the new BM scoring model was thus 0 to 5 points. Distributions of the young infants in our study with bacterial and aseptic meningitis related to the value of our new scoring model are shown in Fig. 1. Infants with none of the above risk predictors were classified as being at very low risk for BM, whereas with any of the above risk predictors were classified as not being at low risk for BM. Using the not low risk criterion (score ≥ 1) with the new prediction model, we identified the young infantile BM with 100% (95% CI 91.9%-100%) sensitivity and 60.3% (95% CI 46.4%-72.9%) specificity (Table 3).

**Table 3:** Application of the new scoring prediction to young infants with cerebrospinal fluid pleocytosis

Infant BM score classification	Bacterial meningitis	Aseptic meningitis
Not low risk (score ≥ 1, n)	44	23
Very low risk (score = 0, n)	0	35
Total	44	58

*N* number

**Fig. 2:**
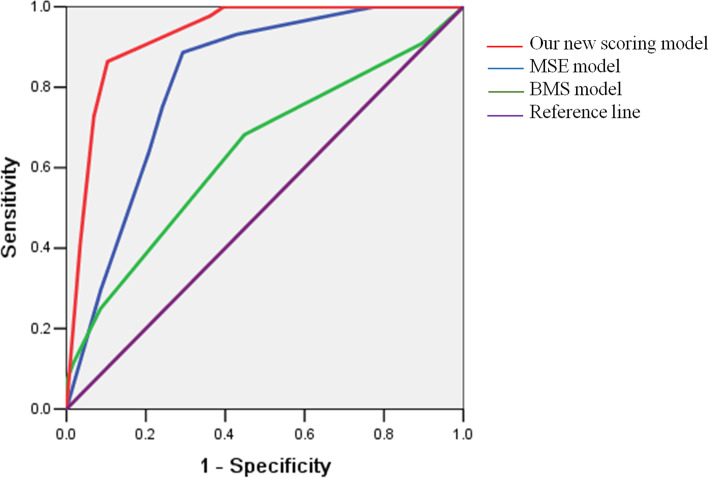
Comparison of the area under the receiver operating characteristic curves of the three scoring models

We also tested BMS model and MSE model in our study, the area under ROC curve was 0.64 (95% CI 0.53–0.75) for BMS model and 0.82 (95% CI 0.74–0.90) for MSE model. Compared with BMS and MSE model, our prediction model had a larger area under ROC curve, and the differences were statistically significant (our model versus BMS, Z = 5.41 *P* < 0.005; our model versus MSE, Z = 3.72 *P* < 0.005). We evaluated the performance of the three models in predicting young infants at not low risk of BM in terms of specificity and sensitivity. Thus, we got 100% (95% CI 91.9%-100%) sensitivity and 60.3% (95% CI 46.6%-72.9%) specificity in our new model, 90.9% (95% CI 78.3%-97.4%) and 10.3% (95% CI 3.9%-21.2%) in the BMS model, 100% (95% CI 91.9%-100%) and 19.0% (95% CI 9.9%-31.4%) in the MSE model, respectively. Our prediction model had a higher specificity than the other two models in our study patients (our model versus BMS, χ^2^ = 547.31 *P* < 0.005; our model versus MSE, χ^2^ = 356.41 *P* < 0.005). The results are shown in Table 4 and Fig. 2.

**Table 4:** Comparison of MSE model, BMS model, and our new scoring model

Models	Area under the ROC curve	95%CI	Sensitivity (%)	Specificity (%)
Our new scoring model	0.93	0.88–0.98	100	60.3
MSE model	0.82	0.74–0.90	100	19.0
BMS model	0.64	0.53–0.75	90.0	10.3

*ROC* receiver operating characteristic, *CI* confidence interval

Finally, we calculated sensitivity and specificity of our new scoring model to identify BM complication. The incidence rate of complication of the BM was 59.1%, which was significantly higher than that of the AM patients (37.9%, χ^2^ = 4.50, *p* = 0.034). Using the not low risk criterion (score ≥ 1) with our new prediction model, we identified complication with 77.1% (95% CI 62.7%-88.0%) sensitivity and 38.9% (95% CI 25.9%-53.1%) specificity.

## Discussion

GBS accounted for a half of cases of BM in young infants in our study, and *Escherichia coli* accounted for about one-third. GBS was predominant in young infants aged 1-3 months in other studies, accounting for 50% of cases from Japan [12], 38% of cases from United Kingdom and Ireland [13], and 32% of cases from Canada [14]. GBS prophylaxis strategies would impact only early-onset (0–6 days of age) GBS meningitis, but not prevent late-onset disease (7–89 days of age) [15, 16]. So there is a need for additional strategies such as GBS vaccines for prevention of late-onset GBS meningitis. In a French survey of *Escherichia coli* meningitis, neonatal cases accounted for 71%, with the 14 days old of the median age at diagnosis [17]. In Japan, there was more non-neonatal *Escherichia coli* meningitis (60%) with the 1 month old of the median age [12]. Unfortunately, we cannot get more details due to the lack of information on neonatal cases. All in all, GBS and *Escherichia coli* remain the most common causes of BM in the young infants.

Generally, it is difficult to distinguish between bacterial and aseptic meningitis at the early stage of the disease, especially in young infants. The majority of infants with CSF pleocytosis receive broad-spectrum antibiotics while awaiting the results of culture tests. Due to the high mortality and morbidity rates of BM [3, 4], it is imperative to receive prompt and appropriate antibiotics to young infants. But some infants with CSF pleocytosis were finally diagnosed as AM, leading to abuse of antibiotics [7]. Therefore, we aimed to establish a new scoring model with 100% sensitivity to detect BM of young infants with CSF pleocytosis, and with high specificity to avoid unnecessary prolonged antibiotic use and excessive hospitalization for infants with AM.

Several diagnostic prediction models have been developed to assess the likelihood of BM in patients presented with suspected central nervous system infection. Up to now, the frequently reported predictors of the BM included seizures; higher level of peripheral WBC count, peripheral ANC, CSF ANC, CSF protein, CRP; lower lever of CSF glucose or CSF/blood glucose ratio [5–9]. However, the cut-offs of the predictors of each model are different, which may be attributed to applicable age difference and racial heterogeneity. Compared with adults and children, young infants have less neck resistance and meningeal irritation due to the incomplete anterior fontanelle closure and poor neck muscle development, but intrcranial hypertension is more commonly manifested as increased anterior fontanelle tension. In addition to CRP, among the currently available diagnostic biomarkers, PCT previously identified and validated as the best biomarker for distinguishing early between BM and AM in pediatric patients [18]. Thus, in the present study, we chose variables the frequently reported predictors above together with AFP and PCT to generate a new scoring model for young infants suspected with BM. As we all know, CSF WBC are composed of multiple nuclear cells and mononuclear cells. In bacterial meningitis, the increase of CSF WBC is mainly due to the increase of neutrophils in multiple nuclear cells, so we chose CSF ANC rather than CSF WBC as a risk factor. The CSF Gram stain result was also not considered in our regression variable, because a positive Gram stain result already indicates BM by itself, a negative test result barely alters the prior odds of BM.

Our new scoring model included PCT ≥ 3.80 ng/ml, CSF glucose ≤ 1.86 mmol/L and CSF protein ≥ 1269 mg/L. PCT as a predictor of BM was also proposed by Dubos and Mintegi [8, 9]. However, most models did not consider PCT but CRP and or ANC (CSF and or peripheral blood) as predictors [5, 6, 19, 20]. In fact, PCT has shown a better performance than traditional markers (CRP, CSF ANC, CSF protein, etc.) to identify invasive bacterial infection, specifically for meningitis [18, 21]. Similarly, the replacement of peripheral ANC with PCT significantly increased the specificity of the BMS model in Garcia’s study [22]. In our study, peripheral WBC count was significantly lower in BM than in AM, but it wasn’t an independent predictor of BM. One possible explanation for peripheral leucopenia in BM group was related to the pathogens causing BM in young infants. Compared with the *Streptococcus pneumonia* and *Haemophilus influenza* in older infants and children, leukopenia was most common in young infants with *Escherichia coli* and GBS [23, 24]. Another possible explanation was that in our study peripheral WBC was perhap*s* obtained earlier than in others’ studies. We reviewed the clinical data and found that 76.5%(78/102)of the infants had peripheral blood routine examination within 24 h of fever. CSF protein concentrations were higher in healthy infants than in older infants and children [25], that was why our CSF protein as a predictor of infantile BM was higher than other models applicable to the wide range of ages of the children [5, 6, 8, 9, 19]. We also found lower levels of CSF glucose was predictive of infantile BM, which was in consistence with Bonus [6].

CSF Gram staining is fast, convenient, and well validated for detecting bacteria. In the BM group, there were 6 infants with the positive Gram-stain, who got the risk score ≥ 4 using our prediction model. Therefore, we recommend that broad spectrum antibiotics should be used to infants with CSF pleocytosis and positive Gram-stain until culture results are available. According to the not low risk criterion (score ≥ 1) of our new model, we identified the young infantile BM with 100% (95% CI 91.9%-100%) sensitivity and 60.3% (95% CI 46.4%-72.9%) specificity. Thus, we also recommend that physicians should give antibiotics to young infants with CSF pleocytosis and risk score ≥ 1.

We also assessed the role of BMS and MSE scoring models in the present study. Their areas under the ROC curve were 0.64 and 0.82, respectively, which were smaller than our new scoring model of 0.93. Most prediction models were developed to accurately identify patients with BM. Because missing BM will have devastating consequences, only 100% sensitivity seems good enough. But, the sensitivity of the BMS model was 90.9%, lower than 100% sensitivity of MSE model and our model. Meanwhile, BMS results were found to be negative for a few children with BM in other studies [26, 27]. That slightly lower sensitivity of BMS model might be partly attributed to the lack of inflammatory indicators such as PCT or CRP as predictors. In addition to emphasizing 100% sensitivity, higher specificity can add value in a clinical setting. On the other hand, the specificity of our model was higher than that of the other two models. The possible reason was that a higher CSF protein level threshold in our model could better discriminate between young infants with BM and those with AM. We also identified complication using the not low risk criterion of our new model, presenting a sensitivity of 77.1% and a specificity of 38.9%, whose performance was less excellent than in identifying the BM in young infants.

Several limitations of our analyses should be considered. First, this was only a retrospective study and was not evaluated in a prospective study. Second, blood glucose before lumbar puncture was not always timely registered, leading to the lack of a more appropriate predictor of CSF/blood glucose ratio. Furthermore, Distribution of main pathogens of BM varies in other regions and would alter the performance of the prediction models. Since our study was realized in a single hospital in an east China population, one may question the generalizability of the findings.

## Conclusion

We presented a new scoring model, appearing sufficiently accurate to permit the timely diagnosis of BM in young infants with CSF pleocytosis. This simple prediction model is more appropriate for young infants and it has a better performance than BMS and MSE models.

## Data Availability

All analyzed datasets are available from the corresponding author upon request.

## Abbreviations

BM: Bacterial Meningitis
BMS: Bacterial Meningitis Score
MSE: Meningitis Score for Emergencies
LP: Lumbar Puncture
CSF: Cerebrospinal Fluid
GBS: Group B Streptococcus
AM: Aseptic Meningitis
AFP: Anterior Fontanel Pressure
WBC: White Blood Cell
ANC: Absolute Neutrophil Count
CRP: C-Reactive Protein
PCT: Procalcitonin
ROC: Receiver Operating Characteristic
IQR: Interquartile Range
CI: Confidence Interval
T: Temperature
